# An oxidation resistant pediocin PA-1 derivative and penocin A display effective anti-*Listeria* activity in a model human gut environment

**DOI:** 10.1080/19490976.2021.2004071

**Published:** 2022-02-01

**Authors:** Taís M. Kuniyoshi, Paula M. O’Connor, Elaine Lawton, Dinesh Thapa, Beatriz Mesa-Pereira, Sara Abulu, Colin Hill, R. Paul Ross, Ricardo P. S. Oliveira, Paul D. Cotter

**Affiliations:** a Biochemical and Pharmaceutical Technology Department, University of São Paulo, São Paulo, Brazil; b Food Biosciences Department, Teagasc Food Research Centre, Moorepark, Fermoy, Co. Cork, Ireland; c APC Microbiome Ireland, University College Cork, Cork, Ireland; d School of Microbiology, University College Cork, Cork, Ireland

**Keywords:** Pediocin PA-1, penocin A, heterologous expression, simulated human distal colon model, anti-*Listeria* activity

## Abstract

Pediocin PA-1 is a class IIa bacteriocin that is particularly effective against the foodborne pathogen *Listeria monocytogenes*. The loss of activity of PA-1 pediocin due to methionine oxidation is one of the challenges that limit the wider application of the bacteriocin. In this study, we heterologously expressed an oxidation resistant form of pediocin PA-1, i.e., pediocin M31L, and compared its activity to that of native pediocin PA-1 and to penocin A, a pediocin-like bacteriocin that displays a narrower antimicrobial spectrum. Minimal inhibitory concentration assays revealed that pediocin M31L was as effective as PA-1 and more effective than synthetic penocin A against *Listeria* with negligible activity against a range of obligate anaerobic commensal gut bacterial species. The anti-*Listeria* activity of these pediocins was also assessed in a simulated human distal colon model assay using the *L. monocytogenes*, spiked at 6.5 ±  0.13 Log CFU/mL, as a bioindicator. At 24 h, pediocin M31L and penocin A (2.6 μM) reduced *Listeria* counts to 3.5 ± 0.4 and 3.64 ± 0.62 Log CFU/mL, respectively, whereas *Listeria* counts were considerably higher, i.e. 7.75 ±  0.43 Log CFU/mL, in the non-bacteriocin-containing control. Ultimately, it was established that synthetic penocin A and the stable pediocin M31L derivative, heterologously produced, display effective anti-*Listeria* activity in a human gut environment.

## Graphical Abstract

**Figure:**
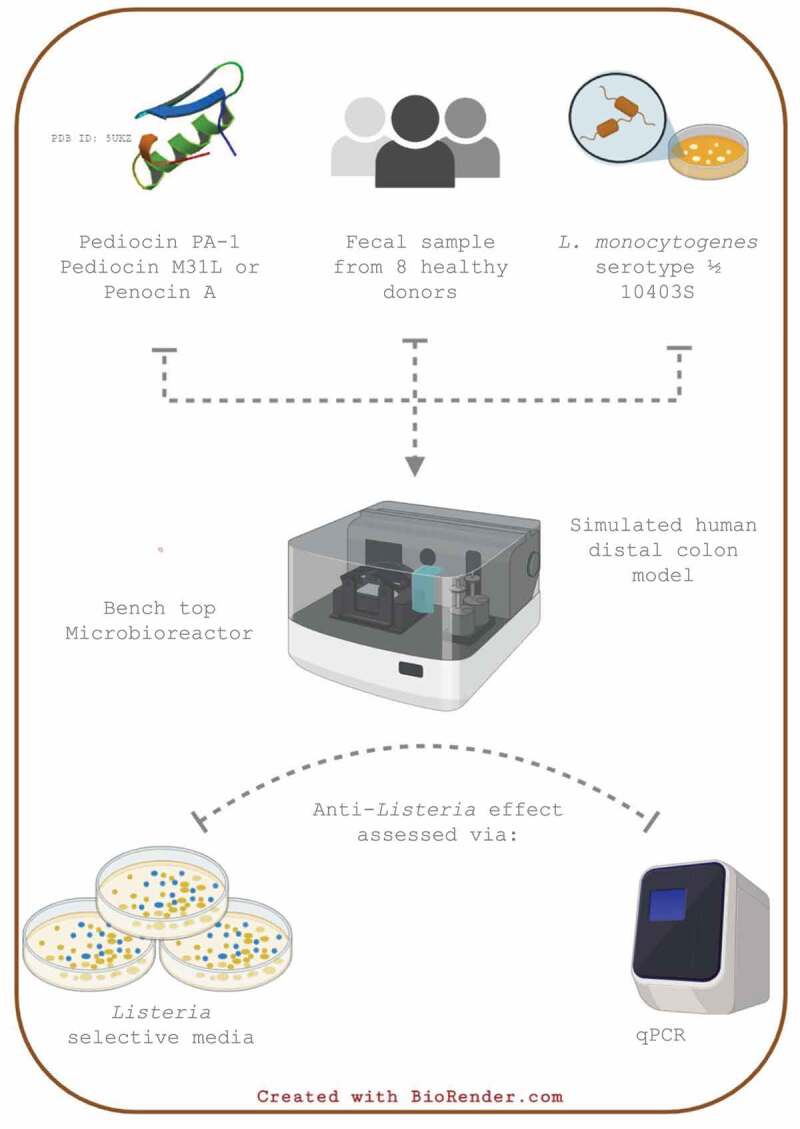

## Introduction

Bacteriocins are a heterogeneous group of antimicrobial peptides ribosomally^1^ produced by certain bacterial strains and are active against a wide range of microbial targets.^2^ Despite their potential for use as food preservatives and decades of research by the food industry, nisin is the only bacteriocin authorized for human consumption by the Food and Drug Administration (FDA) and the European Food Safety Authority (EFSA).^3^ Increasing demand for foods with fewer chemical preservatives combined with the appearance of multi-resistant bacterial strains has led to novel bacteriocins being proposed as an alternative natural food additive, postbiotic components in functional foods or even pharmaceutical therapeutics.^3–5^ The risk of developing antimicrobial resistance can be reduced by using bacteriocins exiguously, and this can be achieved through combining them with other bacteriocins particularly those that use different modes of action, alternative antimicrobials, preservation additives or preservation methods.^4^

Methicillin-resistant strains of *Staphylococcus aureus, Streptococcus pneumoniae, Enterococcus* spp. and *Clostridioides difficile* are, for example, sensitive to the bacteriocin nisin, and its use in combination with conventional antibiotics successfully inhibits an even wider range of Gram-positive pathogens and undesirable bacterial strains.^6,7^ In addition, as certain bacteriocins have been reported as having anti-viral properties, they may have potential for SARS-CoV2 treatment.^8^

Pediocin PA-1 is a class IIa bacteriocin, produced by lactic acid bacteria (LAB) such as *Pediococcus* spp. and *Lactobacillus plantarum*, which exhibits antimicrobial activity against the pathogen *Listeria monocytogenes*.^9–11^ Listeriosis, caused by the Gram-positive bacteria *L. monocytogenes*, is considered one of the most serious foodborne disease due to its high mortality rate, particularly among the elderly, people with compromised immune systems, pregnant women, unborn and neonatal babies.^12^ Contamination is often through ready-to-eat foods where the pathogen can be present in high amounts due to its ability to grow under refrigeration temperatures.^13^ The pathology varies from a noninvasive form (gastroenteritis, febrile diarrhea, headache) to invasive listeriosis, which manifests in more serious symptoms such as septicemia and meningitis. The effectiveness of pediocin PA-1, as an anti-*Listeria* preservative in food, has been studied for decades with many studies using ALTA 2341 (Quest International, Irvine, CA, USA), a commercially available pediocin-containing *P. acidilactici* syrup fermentate. In some studies, ALTA 2341 was incorporated into slurries prepared with Turkey breast^14^ or in “queso blanco”-type cheese^15^ and was found to decrease *L. monocytogenes* counts over time compared to untreated controls. Furthermore, studies have also established that use of pure pediocin led to a reduction of intestinal *Listeria* counts compared to the control in the gastrointestinal tract of BALB/c mice challenged with *L. monocytogenes* LSD 348. This occurred in the absence of major changes in the composition of mouse gut microbiota.^16^ Further investigations using pediocin producers (ped+) or equivalent ped- studies have shown the absence of changes in the overall structure of the fecal microbiota in BALB/c female mice^17^ and in rats with a human-associated microbiota.^18^ This point is important as the bactericidal effect of new therapeutic compounds on the commensal microbiota has attracted more attention as a consequence of the increasing number of diseases and physiological imbalances that have been associated with disruption of the indigenous bacterial community present in the intestine.

An understanding of the dose response of an antimicrobial compound is required for clinical applications. In this regard, the use of pediocin PA-1-producing strains rather than the pure bacteriocin is problematic, given the variety of factors that can influence bacteriocin production in the human gut environment. Large-scale production of pediocin PA-1 in a highly pure form (>95%) is required for cytotoxicity and immunogenicity assessment to facilitate subsequent approval by regulatory authorities. Typically, at least three purification steps are required to produce a sufficiently pure form of a class IIa bacteriocin from a natural producing strain but, unfortunately, yields can often be low (from 350 to 1600 µg of class IIa bacteriocin per liter of culture).^19^ A further complication in pediocin PA-1 production is that the oxidation of methionine at position 31 can significantly reduce its activity, and its substitution to leucine, isoleucine or alanine solve this problem without compromising its anti-*Listeria* activity.^20,21^ To overcome these challenges, in this work, we optimized the production of an oxidation-resistant pediocin PA-1, pediocin PA-1 M31L, employing an expression vector system that controls plasmid copy number in *E. coli* cells previously described by Mesa-Pereira et al.^22^

Chemical synthesis of penocin A, another bacteriocin that harbors a YGNGVX_1_CX_2_K/NX_3_X_4_C, (X_1–4_:polar uncharged or charged residues) pediocin box in its sequence was carried out in tandem to compare its antimicrobial efficacy against *L. monocytogenes* and the commensal gut microbiota. Penocin A is considered a silent bacteriocin as its mature form is not produced naturally due to the absence of a key gene(s) in the associated natural strains. Diep et al.^23^ revealed that heterologous expression of the pediocin inducer factor led to penocin A expression in *Pediococcus pentosaceus* 25745 and its inhibition rate against 71 strains from a collection of 8 Gram positive bacteria genera (*Carnobacterium, Clostridioides, Enterococci, Lactobacilli, Lactococci, Leuconostoc, Listeriae, Pediococci*) was lower (48%) than that presented by pediocin PA-1 (58%). This reduced antimicrobial activity against some important bacterial groups (e.g *Lactobacilli, Lactococci, Leuconostoc)* may be a desirable feature when used as a therapeutic agent. Here the anti-*Listeria* effects of pure forms of recombinant pediocin M31L, natural pediocin PA-1 and synthetic penocin A were each evaluated in an *ex vivo* model that simulates the human gut environment for the first time. This study verifies the efficiency of the pediocin variants against *L. monocytogenes* and highlights the merits of progressing to *in vivo* experiments.

## Results

### Cloning and expression of pediocin M31L

**Figure 1.**
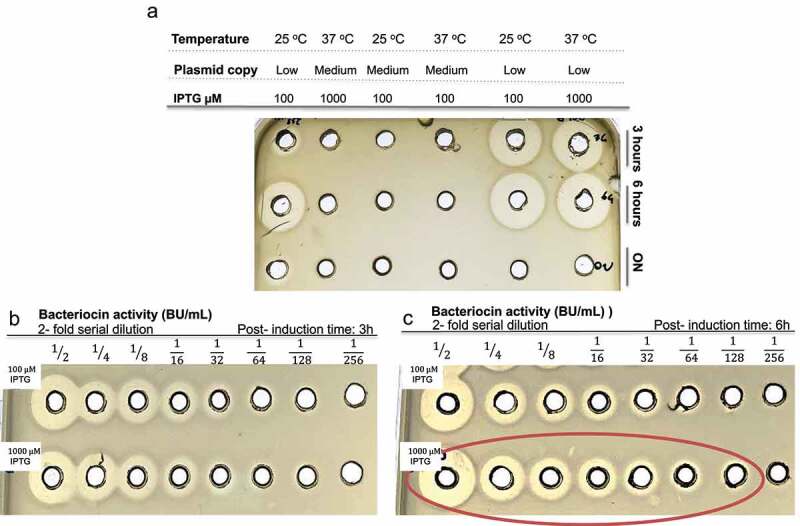
Antimicrobial activity of cell-free supernatant (CFS) produced by *E. coli*/pETcoco-pedM31L against *L. innocua* DPC3572 in BHI media. Optimization of heterologous expression was assessed under different conditions including plasmid copy number [low (L) vs. medium(M)], temperature (25 °C vs. 37 °C), IPTG concentration (100 μM vs. 1000 μM) and period of induction (3 h vs. 6 h vs. overnight- ON) (a) bacteriocin activity (BU/mL) produced by recombinant cells at 100 μM (top) and 1000 μM (bottom) at 3 hours (b) and 6 hours (c). Relative quantification of cell-free supernatant (BU/mL). Optimum conditions were low plasmid copy number induced with 1mM of IPTG for 6 hours at 37 °C (red circle). The data shown is representative of two independent assays.

Recombinant expression of pediocin M31L was evaluated indirectly through antimicrobial activity of culture supernatant against *Listeria innocua* DPC3572. It was established that pediocin M31L was produced under low plasmid copy number following induction for 3 and 6 hours but not overnight as observed by the well-defined inhibition zones (Figure 1a). To quantify the highest anti-*Listeria* activity, samples with the largest inhibition diameters were serially diluted two-fold to quantify Bacteriocin Units (BU) (Figure 1b and 1c). Higher antimicrobial activity within the cell-free supernatant (CFS) was achieved in the presence of 1000 μM of IPTG following 6 hours induction at 37°C, the only condition that achieved 1/128 BU (Figure 1c- red circled). This assay was performed in two independent assays. In addition, it was previously demonstrated that no anti-*Listeria* activity was observed in the extracellular fraction of *E. coli* (DE3) Tuner^TM^ transformed with modified pETcoco empty vector.^22^ Pediocin M31L and native pediocin PA-1 were purified from cell-free supernatant of *E. coli*/pETcoco-pedM31L and *P. acidilactici* LMG 2351, respectively, in a 5-step process using Amberlite XAD16N beads, SP sepharose, C18 SPE and two RP HPLC purification steps with a yield of 0.725 mg per liter of *E. coli*/pETcoco-pedM31L culture and 0.625 mg per liter of *P. acidilactici* LMG 2351 culture. Purity of >95% for pediocin PA-1 (˜4624 Da) and M31L (˜4007 Da) were confirmed by MALDI-TOF mass spectrometry (data not shown).

### Comparison of pediocin PA-1, pediocin M31L and penocin A antimicrobial activity

**Figure 2.**
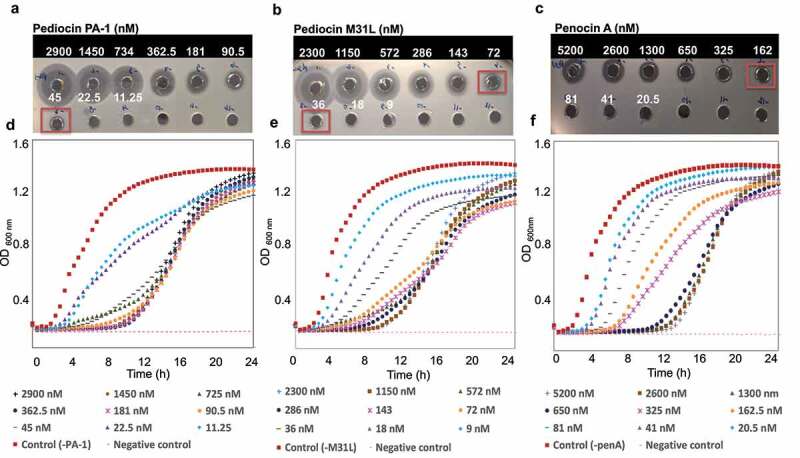
Antimicrobial activity of pure pediocin PA-1 (a), pediocin M31L (b), and penocin A (c) in 0.8% BHI agar or BHI broth (d,e,f, respectively) in the presence of an initial inoculum of ~6.5 Log CFU/mL of *L. monocytogenes* 10403S. Initial bacteriocin concentrations of 5.85 µM of pediocin PA-1, 4.6 µM of pediocin M31L and 10.4 µM of penocin were diluted 2 fold in sterile PBS buffer, and their MIC against Listeria (red square) were evaluated in both soft BHI agar (a,b,c) and BHI broth (d,e,f) for 24 h at 37 °C, OD600 was read at 30 min intervals. The data shown is representative of three independent assays.

The effect of pure pediocin PA-1, pediocin M31L and penocin A on *L. monocytogenes* 10403S was evaluated in BHI soft agar (0.8% agar) (Figure 2d,e,f,) and BHI broth over 24 h at 37 °C (Figure 2d,e,f,). Figure 2 shows the average of n = 3 independent biological replicates where MIC of pediocin PA-1 was 45 nM (Figure 2a), pediocin M31L was 72–36 nM (Figure 2b) and penocin A was 162 nM (Figure 2c) in BHI soft agar containing 10403S cells. In broth, the bacteriocins were bacteriostatic, with an extended lag phase in the presence of the lowest concentration tested for all bacteriocins relative to the bacteriocin free control. The delay in the initiation of exponential *Listeria* growth >8 hours in comparison with bacteriocin-free control was obtained with 90–45 nM nM of Pediocin PA-1, 72 nM of Pediocin M31L and 650 nM of penocin A (Figure 2d,e,f,, respectively). These conditions showed significantly different OD_600nm_ compared to the bacteriocin-free control at OD_600nM_ of 0.4 (one-way ANOVA followed by Tukey’s post hoc test *p* < .05).

**Figure 3.**
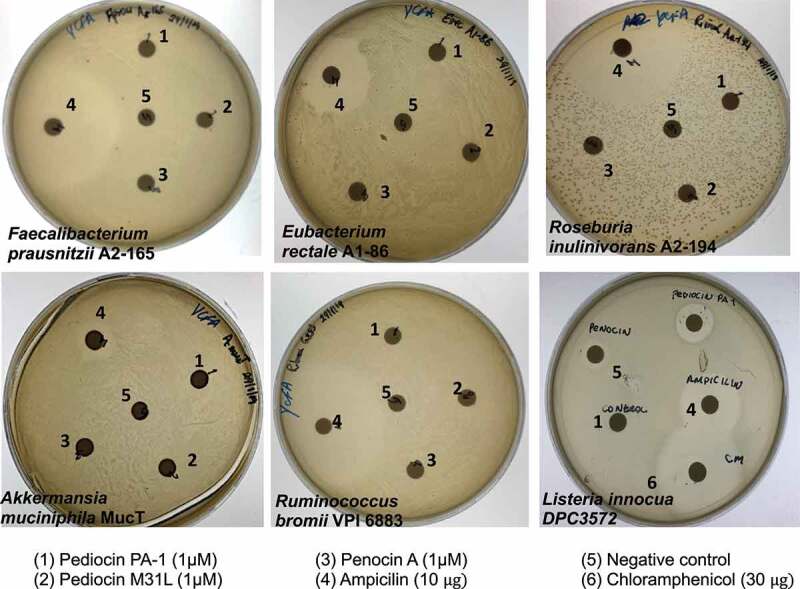
The effect of pediocin PA-1, pediocin M31L and penocin A on human gut commensal bacterial strains. Filter discs containing 1µM of each bacteriocin were tested against *Faecalibacterium prausnitzii* A2-165, *Eubacterium rectale* A1-86, *Roseburia inulinivorans* A2-194, *Akkermansia muciniphila* MucT, *Ruminococcus bromii* VPI 6883 and *L. innocua* DPC 3572. Ampicillin (10 µg) and chloramphenicol (CM) (30 µg) were used as a control. The data shown is representative of two independent assays.

The antimicrobial activity of these bacteriocins against selected human gut commensal strains was also evaluated. *Faecalibacterium prausnitzii* A2-165, *Eubacterium rectale* A1-86, *Roseburia inulinivorans* A2-194, *Akkermansia muciniphila* MucT and *Ruminococcus bromii* VPI 6883, originally isolated from human feces,^24^ have been used as indicators to elucidate if bacteriocins effective against *L. monocytogenes* have a detrimental effect on the commensal microbes. These bacteria are abundant species in a normal healthy gut and have also gained special interest as next generation health-promoting species.^25^ No inhibition was observed against these strains when 1 µM of pediocin PA-1, 1 µM pediocin M31L and 1 µM penocin A were tested (Figure 3). Antimicrobial activity was observed against the control strain, *L. innocua* DPC3572, in the presence of 1 µM of pediocin PA-1, 1 µM pediocin M31L and 1 µM penocin A. In addition, ampicillin (10 µg) or chloramphenicol (30 µg) resulted in a zone of inhibition against all strains assessed. This assay was performed in two independent assays.

### *Ex vivo* model assays

**Figure 4.**
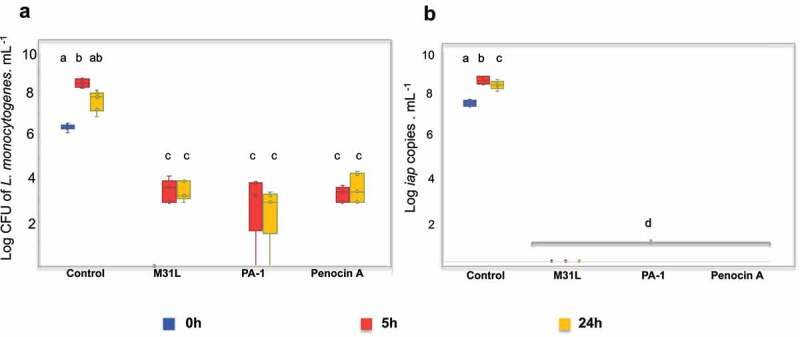
*Listeria monocytogenes* quantification on selective media Log CFU/ mL (a) and via qPCR (b). Viable *L. monocytogenes* cells were assessed by plating *ex vivo* fermentation samples on *Listeria* selective media at T0h, T5h and T24h (a). A specific region of iap gene was amplified from all samples in real time PCR (b). Values are expressed as mean for two independent assay (n=5). Two way ANOVA followed by Tukey’s post- hoc test were performed. Different superscript letters indicate statistically significant difference (*p* < 0.05). The data shown is representative of two independent assays.

The ability of natural pediocin PA-1, recombinant M31L and synthetic penocin A to inhibit *L. monocytogenes* 10403S in a simulated human gut environment was assessed using 6.5 Log CFU/mL of the bioindicator strain inoculated in fecal media and slurry and fermented in a bench top bioreactor. *Listeria* numbers increased from 6.5 ±  0.13 Log CFU/mL (T0h) to 8.56 ±  0.16 Log CFU/mL and 7.75 ±  0.43 Log CFU/mL after 5 h and 24 h of fermentation, respectively, in the absence of bacteriocins (positive control; Figure 4a). The addition of penocin A, pediocin PA-1 or pediocin M31L at 2.6 µM led to a complete inhibition of *Listeria* cells at time 0 h, i.e., immediately after addition of the bacteriocins. A small quantity of viable *Listeria* cells was detected in the presence of pediocin M31L (3.5 ±  0.47 and 3.5 ±  -0.4 Log CFU/mL), pediocin PA-1 (3.01 ±  1.5 and 2.5 ±  1.29 Log CFU/mL) or penocin A (3.3 ±  0.32 and 3.64 ±  0.62 Log CFU/mL) at T5h and T24h, respectively.

qPCR using primers to amplify a variable region of *iap* gene (Figure 4b) were also used to quantify *Listeria*. This gene encodes p60 extracellular protein produced by all *Listeria* species, but a specific region of the gene can be used to differentiate between *L. innocua* and *L. monocytogenes* species.^26^ Figure 4b shows that the Log of *iap* copy number/mL increased from 7.58 ±  0.126 Log *iap* copy/mL (T0h) to a mean of 8.67 ±  0.17 Log *iap* copy/mL after 5 h of fermentation and subsequently reduced to 8.45 ±  0.18 Log *iap* copy/mL (T24h) in the bacteriocin-free control. In the presence of pediocin PA-1, the recombinant M31L and penocin A, *iap* amplification was not detected. It is important to highlight that the detection limit of this qPCR assay is 4.5 Log *iap* copy/mL.

## Discussion

Heterologous expression of pediocin PA-1 has been achieved previously in different *E. coli* strains (BL21 (DE3), M15/pRep4, Origami (DE3), V850) but, to our knowledge, significant quantities of peptide were not obtained in a pure form from *E. coli*.^27,28^ Despite the ability to obtain recombinant pediocin using affinity chromatography, a further purification step to remove the fusing protein or HIS-tag results in a considerable loss of bacteriocin yield typically, i.e. 0.06 mg per 100 mL of culture.^28^ Essential genes, necessary for the heterologous expression of mature pediocin in the extracellular fraction in *E. coli*, were identified by Mesa-Pereira et al.^22^ However, pediocin PA-1 production was considerably lower compared to that obtained by the natural producer *P. acidilactici* LMG2351, probably due to oxidation of recombinant pediocin PA-1 as evidenced by a second peptide mass of ~4640 Da.

Pediocin PA-1 instability due to methionine oxidation has been widely reported.^20,21,29^ Indeed, Johnsen et al.^20^ demonstrated for the first time that substituting methionine at position 31 to leucine, isoleucine or alanine prevents peptide oxidation and improves its stability. Indeed, a synthetic pediocin PA-1 analog with leucine instead of methionine (M31L) showed the same antimicrobial activity as the original peptide.^20^ Therefore, in this study, a pediocin M31L derivative was expressed for the first time in *E. coli*. This microorganism is widely used for heterologous expression due to the availability of well-established molecular tools and the ability to grow *E. coli* in numerous inexpensive and/or mineral media, thereby minimizing costs.^30^ The yield of pediocin M31L (0.725 mg/Liter of culture supernatant) obtained in this study with >95% of purity is reasonable when compared with other bacteriocin studies,^19^ and there is potential to further improve yield. It is worth noting that scale up of pediocin M31L production in *E. coli* requires further evaluation taking into account cost benefit analysis and other variables such as substrate selection. Indeed, mineral media is often used for *E. coli* heterologous expression and could lead to a reduction in the number of purification steps, potentially increasing yield as mature pediocin is present in the supernatant fraction.

In this study, pediocin M31L displayed similar anti-*Listeria* activity to native pediocin PA-1. When the antimicrobial activity of the three pediocins was assessed against *Listeria* in broth media, a bacteriostatic effect was observed even at higher bacteriocin concentrations. Mechanisms of bacteriocin resistance and bacteriocin tolerance are not fully studied but have been described within all classes of bacteriocin.^31^ Bacteriocin resistance can be both acquired and innate, and the main resistance mechanism for class IIa bacteriocins is the downregulation of the expression of the mannose phosphotransferase system (Man-PTS), which has been described for *E. faecalis* and *L. monocytogenes*.^32–34^ The regulatory gene *rpoN* also influences the *mpt* expression and consequently influences the development of resistance.^35^

Metabolism shifts have also been observed when pediocin PA-1-sensitive and -resistant *E. faecalis* were compared^36^ and with *L. monocytogenes* bacteriocin-resistant strains.^37,38^ Upregulation of genes involved in the usage of sugars may play a role in bacteriocin resistance, and recently, it has shown that cellobiose and sucrose increased the generation of resistant *L. monocytogenes* when compared to other carbohydrates tested.^39^ Additionally, the composition of the food matrix should be considered as it could favor the development of bacteriocin resistance and at the same time modify the sensory properties of the product by inducing the growth of the spoilage bacteria.

Undoubtedly, studies carried out with the pure form of these bacteriocins lead to more accurate assessments of the dose needed to observe an anti-*Listeria* effect than those carried out with bacteriocin containing fermentates or bacteriocin-producing cultures. The greater precision in the amount of antimicrobial compound required for efficacy is essential to design more effective and reproductive protocols, reducing the chances of bacteriocin-resistant strain appearance. Certainly, more controlled use of emerging food additives will be increasingly requested for regulatory agencies.

The antimicrobial activity of bacteriocins on gut commensal bacteria is also of critical importance as strains from species such as *A. muciniphila, F. prauznitzii* and *Eubacterium* spp. have gained attention due to their health-promoting effects and the potential to serve as next-generation probiotics or biotherapeutics.^40^ In the present study, none of the pediocin variants tested displayed activity against commensal bacteria, though undoubtedly further experiments should be carried out using encapsulated pediocins in controlled clinical studies. Previous investigation whereby CFS from LAB strains containing nisin A, nisin Z or pediocin PA-1^41^ were assessed against different commensal bacteria. While both nisin variants displayed activity against known commensal bacteria such as *Eubacterium biforme* DSM 20477, *Ruminococcus productus* DSM 2950 and many species of *Bifidobacterium* and *Lactobacillus*, none of the tested strains were inhibited by pediocin PA-1, indicating that this bacteriocin might be a more appropriate treatment for gut infections as no disturbance of the intestinal bacterial balance is likely to occur.

Currently, no bacteriocin is used to treat gut bacterial infections despite the unquestionable evidence of their efficacy. Simulated gut model assays performed, at a concentration of 2.6 µM of pediocin PA-1, pediocin M31L or penocin A, resulted in total inhibition of *Listeria* after 5 hours of fermentation, and, even after 24 h of fermentation, *Listeria* counts were considerably lower (>4 Log CFU/mL) than the control. In this regard, it is noteworthy that Dabour et al.^16^ observed in a mice model challenged with *L. monocytogenes* LSD348 that three intragastric doses of pure pediocin PA-1 (250 μg) led to almost a 2 log reduction in *Listeria* counts in fecal samples, while only a slight anti-*Listeria* effect was observed when the pediocin producer strain *P. acidilactici* UL5 was administrated, reinforcing the importance of antimicrobial concentrations and potential challenges associated with the use of producing strains in treating a specific pathogen.

Undoubtedly, there are a number of hurdles to overcome with respect to the use of these pediocins for clinical applications. In this work, the use of a microbioreactor to simulate colon conditions proved to be a very reproducible and reliable platform to verify the listericidal effect of the studied bacteriocins. Encapsulation employed can protect the bacteriocins from protease breakdown in the gastrointestinal tract, while combining pediocins with other antimicrobial compounds also merits consideration. The evaluation of different pediocin carriers (nanocrystals and nanofibers of cellulose or nanostructured lipid) with different dosages of pediocin still needs to be tested prior to *in vivo* and clinical trials. The use of 24-well microbioreactors has been widely applied^42,43^ and would accelerate the optimization of pediocin as a therapeutic product. Regulating the use of a new drug is a very complex and laborious process, and optimizing the production of a more stable form of pediocin and applying it to initial *ex vivo* model studies was a notable achievement in this study and forms the basis of future more targeted clinical studies.

## Materials and methods

### Bacterial strains, culture conditions and plasmid constructions

The pediocin PA-1 structural gene (*pedA*), containing a codon change to introduce a methionine to leucine substitution at position 31 (M31L), and the associated accessory (*pedC*) and transport genes (*pedD*) were synthesized (Eurofins MWG Operon Inc., Ebersberg, MU, Germany) with codons optimized for expression in *E. coli*. This DNA fragment was amplified by polymerase chain reaction (PCR) using Platinum® Taq DNA Polymerase (Thermo Fisher Scientific) and primers containing *Sph*I and *Avr*II restriction sites (5ʹ-3ʹCCTGCATGCAAATGAAGAAAATCGAAAAGC and CATACCTAGGCTAGGTCACTCCTGATTATGA). PCR-generated fragments and the modified pETcoco-2 expression vector^22^ (Novagen, EMD Millipore, Billerica, MA, USA) were digested with *Sph*I and *Avr*II, and the resulting fragments were ligated with T4 DNA ligase (Thermo Fisher Scientific). One-shot TOP10 cells (derivatives of *E. coli* DH10β) were used for standard cloning procedures, and transformants containing pETcoco-pedM31L were confirmed by PCR amplification and sequencing. PCR clean up steps and plasmid extractions were performed with NucleoSpin® Extract II Kit and NucleoSpin® plasmid kits (Macherey-Nagel, Duren, CO, Germany), respectively.

*E. coli* (DE3) Tuner^TM^ cells (Novagen, EMD Millipore) were transformed with pETcoco-pedM31L, and the recombinant expression was carried out in Luria-Bertani (Difco Laboratories, Detroit, MI, USA) medium supplemented with ampicillin (50 μg/mL). Cells were grown to OD_600nm_ 0.5–0.6 and were subjected to different temperatures (25 °C or 37 °C), isopropyl β-d-1-thiogalactopyranoside (IPTG) (Sigma-Aldrich Inc., Arklow, Ireland) concentrations (100 or 1000 μM) and periods of induction (3 h, 6 h or overnight). Plasmid copy number was controlled by the addition of 0.2% of D(+) glucose (Sigma-Aldrich Inc.) for low copy state or 0.01% L-+-Arabinose (Sigma-Aldrich Inc.) for medium copy state. To determine antimicrobial activity, *E. coli*/pETcoco-pedM31L supernatants were collected by centrifugation at 10,000 x g for 15 min at 10°C and assessed against *Listeria innocua* DPC3572 via the well-diffusion method. Quantification of pediocin antimicrobial activity was measured according to Mesa-Pereira et al.^22^

The minimal inhibitory concentrations of pure (>95%) pediocin PA-1, pediocin M31L or penocin A were assessed in soft media (0.8% agar) or broth Brain Heart Infusion (BHI) media using *Listeria monocytogenes* serotype 1/2a 10403S as a bioindicator. Initial bacteriocin concentrations were determined by QuantiPro™ BCA Assay Kit (Sigma-Aldrich Inc.) and then serially two-fold diluted in 0.1% trifluoroacetic acid (TFA). The resulting samples were assessed in BHI soft media or broth containing ~10^6^ CFU 10403S cells (~10^6^ CFU). In soft media, 50 μL bacteriocin-containing samples were added to each well (5 mm diameter) and incubated at 37 °C for 24 h, whereas in broth, *Listeria* growth was monitored at 600 nm every 30 min at 37 °C for 24 h using a Synergy HT plate reader (BioTek Instruments Inc, Winooski, VT, USA). The antimicrobial activity of these pediocins against specific human gut commensal strains was evaluated in anaerobic conditions (80% N_2_, 10% H_2_, 10% CO_2_ gas mixture) in pre-reduced agar form of YCFAGSC medium.^44^

### Purification of pediocin PA-1 from *P. acidilactici* LMG 2351 and pediocin M31L from recombinant *E. coli*

Pediocin PA-1 and pediocin M31L were purified as follows: 800 mL of *P. acidilactici* LMG 2351, a natural pediocin PA-1 producer,^45^ or *E. coli*/pETcoco-pedM31L cells were harvested by centrifugation at 10,000 x g for 15 min at 10 °C and the supernatant loaded onto an Econo column containing 60 g Amberlite XAD16N beads (Phenomenex, Macclesfield, CH, UK) prewashed with Milli Q water. The column was washed with 250 ml 30% ethanol and antimicrobial activity eluted with 250 ml 70% propan-2-ol (IPA) 0.1% TFA. To perform the ion exchange purification step, the IPA was removed from the eluent sample via rotary evaporation and the pH adjusted to approximately 4.4. The resulting sample was applied to an Econo column containing 60 ml SP-Sepharose beads (GE Healthcare, Bio-sciences AB, Uppsala, UP, SWE) pre-equilibrated with 250 ml 20 mM sodium acetate pH 4.4. The column was washed with 60 ml of 20 mM sodium acetate pH 4.4 and antimicrobial activity eluted with 250–300 ml 20 mM sodium acetate pH 4.4 containing 1 M NaCl. The salt-containing eluent from the SP-sepharose column was applied to a Strata–E C18 SPE column (Phenomenex) pre-equilibrated with methanol and water. The column was washed with 25% ethanol and antimicrobial activity eluted with IPA.

The sample was further purified by two Reversed Phase High Performance Liquid Chromatography (RP-HPLC) steps. The IPA was removed from the C18 SPE eluent by rotary evaporation and applied to a semi-preparative Jupiter Proteo (10 x 250 mm, 90 Å, 4 µm) RP-HPLC column (Phenomenex) running a 25–40% acetonitrile 0.1% TFA gradient. The eluent was monitored at 214 nm, and fractions were collected at 1-minute intervals. HPLC fractions were tested against *L. innocua* DPC3572, and the fractions with the highest antimicrobial activity were submitted to a final purification step on the same semi-preparative RP-HPLC column with a shallower 30–40% acetonitrile gradient. Fractions deemed pure by MALDI-TOF mass spectrometry (Shimadzu Biotech, Manchester, CO, UK) were pooled and lyophilized (Genevac, Ipswich, SU, UK).

Penocin A was synthesized on a H-Arg(PBF)-HMPB-ChemMatrix® resin (PCAS Biomatrix Inc., Saint-Jean-sur-Richeliieu, QC, CA) by microwave-assisted solid-phase peptide synthesis (MW-SPPS) performed on a Liberty Blue microwave peptide synthesizer (CEM Corporation, Mathews, NC, USA). Crude peptide was purified using a Semi Preparative Jupiter Proteo RP-HPLC column described above (Phenomenex). Fractions containing the desired molecular mass were identified by MALDI-TOF mass spectrometry and were pooled and lyophilized on a Genevac HT 4X lyophilizer (Genevac Ltd.).

### Human distal colon model assays

Distal colon model assays were approved by the Clinical Research Ethics Committee of the Cork Teaching Hospitals (protocol no. APC 104). Human fecal inoculum was prepared as previously described by O´Donnell et al.^42^ Briefly, each standardized fecal inoculum consisted of eight healthy donors who had not taken an antibiotic for 6 months prior to donating a fecal sample and did not harbor any significant acute or chronic illness. *Ex vivo* fermentation was carried out in bench top bioreactors micro-Matrix (Applikon Biotechnology, Heertjeslaan, DE, NE) according to the protocol described by O´Donnell et al.^42^ for each well: 400 µL of slurry, 6.5 log CFU/mL of *L. monocytogenes* 10403S, 2.6 µM of purified pediocin PA-1, pediocin M31L or penocin A were inoculated in fecal media^46^ up to a final volume of 5 mL. A well containing the bioindicator strain alone (no bacteriocin) was used as a control. Each treatment was performed in two independent assays. Samples were collected during the fermentation at time 0 (T0h), 5 (T5h) and 24 hours (T24h) and were serially diluted in maximum recovery diluent (MRD) (Oxoid Ltd., Basingstoke, HA, UK) and plated on *Listeria* Chromogenic Media supplemented with Polymyxin and Ceftazidime (Cruinn Diagnostics Ltd., Dublin, DU, IRE) at 30 °C for 48 h.

For total DNA extraction, 1 mL of fermentation sample was extracted using QIAmp Power Fecal DNA Kit (QIAGEN, Crawley, UK) according to the manufacturer’s recommendation. Presence of *Listeria* was quantified through qPCR using *iap* as a target as described by Hein et al.^26^ For statistical significance analysis, two-way ANOVA followed by Tukey’s *post hoc* tests were performed using Minitab 17 Statistical Software (Minitab Inc., State College, PA, USA) (www.minitab.com).

## Data Availability

The data that support the findings of this study are openly available in figshare.com at http://doi.org/10.6084/m9.figshare.16908805.
